# Uncovering the gene regulatory network of type 2 diabetes through multi-omic data integration

**DOI:** 10.1186/s12967-022-03826-5

**Published:** 2022-12-16

**Authors:** Jiachen Liu, Shenghua Liu, Zhaomei Yu, Xiaorui Qiu, Rundong Jiang, Weizheng Li

**Affiliations:** 1 Department of General Surgery, Third Xiangya Hospital Central South University, No. 138 Tongzipo Road Yuelu District, Changsha, 410013 Hunan People’s Republic of China; 2 Department of Thyroid and Breast Surgery, The Frist Afflicted Hospital of Fujian Medical University, No. 20 Chayzhong Road, Taijiang District, Fuzhou, 350005 Fujian People’s Republic of China; 3 Xiangya Medical College, Central South University, No. 138 Tongzipo Road Yuelu District, Changsha, 410013 Hunan People’s Republic of China; 4 The Center of Systems Biology and Data science, School of Basic Medical Science, Central South University, Changsha, Hunan People’s Republic of China

**Keywords:** Multi-omics network, Key driver genes, Type 2 diabetes, Drug repositioning, Bioinformatics analysis

## Abstract

**Background:**

Type 2 diabetes (T2D) onset is a complex, organized biological process with multilevel regulation, and its physiopathological mechanisms are yet to be elucidated. This study aims to find out the key drivers and pathways involved in the pathogenesis of T2D through multi-omics analysis.

**Methods:**

The datasets used in the experiments comprise three groups: (1) genomic (2) transcriptomic, and (3) epigenomic categories. Then, a series of bioinformatics technologies including Marker set enrichment analysis (MSEA), weighted key driver analysis (wKDA) was performed to identify key drivers. The hub genes were further verified by the Receiver Operator Characteristic (ROC) Curve analysis, proteomic analysis, and Real-time quantitative polymerase chain reaction (RT-qPCR). The multi-omics network was applied to the Pharmomics pipeline in Mergeomics to identify drug candidates for T2D treatment. Then, we used the drug-gene interaction network to conduct network pharmacological analysis. Besides, molecular docking was performed using AutoDock/Vina, a computational docking program.

**Results:**

Module-gene interaction network was constructed using MSEA, which revealed a significant enrichment of immune-related activities and glucose metabolism. Top 10 key drivers (*PSMB9, COL1A1, COL4A1, HLA-DQB1, COL3A1, IRF7, COL5A1, CD74, HLA-DQA1,* and *HLA-DRB1*) were selected by wKDA analysis. Among these, *COL5A1, IRF7, CD74*, and *HLA-DRB1* were verified to have the capability to diagnose T2D, and expression levels of *PSMB9* and *CD74* had significantly higher in T2D patients. We further predict the co-expression network and transcription factor (TF) binding specificity of the key driver. Besides, based on module interaction networks and key driver networks, 17 compounds are considered to possess T2D-control potential, such as sunitinib.

**Conclusions:**

We identified signature genes, biomolecular processes, and pathways using multi-omics networks. Moreover, our computational network analysis revealed potential novel strategies for pharmacologic interventions of T2D.

**Supplementary Information:**

The online version contains supplementary material available at 10.1186/s12967-022-03826-5.

## Introduction

Type 2 diabetes (T2D) is a chronic metabolic disease distinguished by insulin resistance and elevated blood glucose levels. As a global endemic, recent data from the Centers for Disease Control and Prevention (CDC) [1] suggested that as of 2019, roughly 28.7 million people in the United States (8.7% of the total U.S. population) were diagnosed with diabetes, of which about 90–95% have T2D. As prolonged hyperglycemia is a high-risk factor for heart disease, chronic kidney disease (CKD), and nerve damage, T2D imposes a substantial economic burden on society [2]. In addition, calculations based on epidemiological data suggested that the expenditure on diabetes in the U.S. was approximately $327 billion in 2017, including $237 billion in direct medical costs and $90 billion in lost productivity [3].

Due to the complexity of the onset and progression of T2D and the tandem with various diseases, analysis from multiple levels could exponentially augment our understanding of its pathophysiological mechanism. Including genomics, epigenomics, transcriptomics, etc., multi-omics analysis [4] can provide a list of disease-related differences that can be used as biomarkers of the disease process and uncover critical pathways in disease. For instance, Yang-Tay et al. discovered the DNMT1-NT5C2-insulin receptor pathway using the DNA methylation array data, demonstrating that DNMT1 is relevant to the susceptibility of T2D patients [5]. Besides, through the single-cell transcriptome analysis, Lawlor et al. revealed that PP/gamma cells in T2D patients could integrate central and peripheral hunger and satiety signals, which increases our accurate knowledge of the molecular components of rare islet cells [6]. However, unlike single-omics studies, multi-omics studies can be more comprehensive and accurate. At present, multi-omics studies on T2D have received extensive attention. For example, to better understand preT2D status, Wenyu et al. [7] conducted a cohort study of 106 normal individuals and individuals with prediabetes. Through conjoined analysis of transcriptomic and proteomic data, etc., the study indicated a new sight in that the gut microbiota of insulin-resistant individuals may have a decreased response to respiratory viral infection. This could lead to the emergence of chronic inflammation, which promotes the progression of T2D in preT2D patients. In addition, by combining GWAS data with other multi-omics datasets, Yon Jung [8] revealed the common pathways shared by IGF-I and IR, such as glycosaminoglycan biosynthesis, etc. It provided assistance for a more comprehensive understanding of the molecular mechanism of the IGF-I/IR axis. Although previous observations are of great significance, by comprehensively using the data of genomics, transcriptomics, and epigenomics to explore disease-related gene expression signatures, our cognition of T2D and the clinical transformation of drug discovery can both be advanced.

**Fig. 1:**
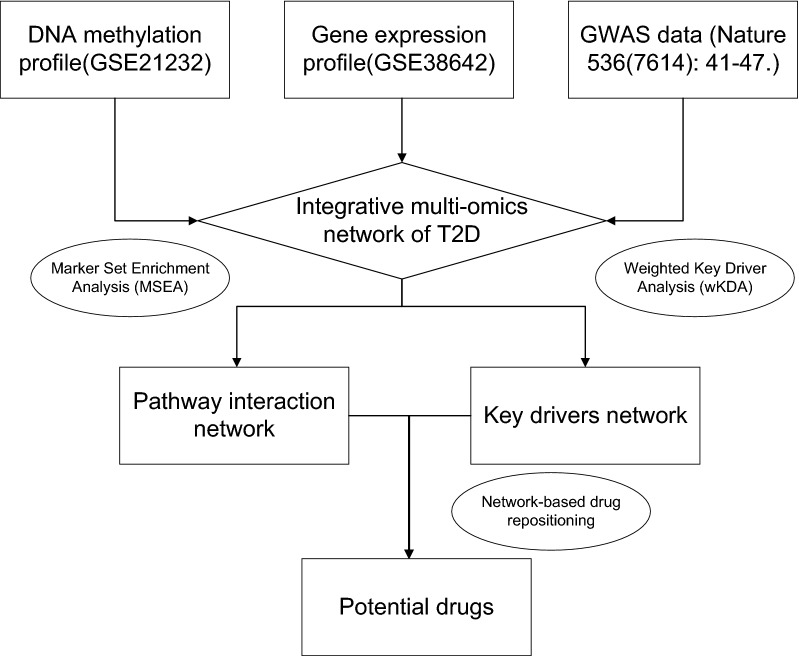
The outline of the analysis pipeline. The analysis pipeline of the study is shown graphically to increase organization and readability

In this study, more accurate potential key genes and their regulatory mechanisms in T2D were identified by constructing gene regulatory network through multi-omics analysis, which contributes to demonstrating pathology, and identifying drug targets of T2D (Fig. 1).

## Methods

### Data source

Gene expression profiles and DNA methylation profiles of T2D were filtrated through the Gene Expression Omnibus (GEO) database (http://www.ncbi.nlm.nih.gov/geo). Inclusion criteria were as follows: (1) Availability of islets from T2D patients in the dataset; (2) Ten or more islet samples in the dataset. Three eligible datasets were selected, including GSE38642 and GSE21232 (training set), and GSE25724 (test set). Additionally, genomics data (Nature 536(7614): 41–47.) was retrieved from Mergeomics web server. The details of the data are shown in Table 1.

**Table 1:** Basic information of selected datasets

Dataset	Platform	Tissue (Homo sapiens)	Samples (number)			Experiment type	Attribute	Author/reference
			Total	T2D	Non-T2D			
GSE38642	GPL6244	Human islets	30	9	21	Array	Test	Taneera [56]
GSE25724	GPL96	Human islets	13	6	7	Array	Validation	Dominguez [57]
GSE21232	GPL8490	Human islets	16	5	11	Array	Test	Volkmar [58]
Nature 536(7614): 41–47		Human	2657	1326	1331	Array	Test	Fuchsberger [59]

### Data process

All microarray data were submitted to the GEO database (http://www.ncbi.nih.gov/geo). The raw data were downloaded as MINiML files. It contains the data for all platforms, samples, and GSE records. The extracted data were normalized by log2 transformation. The microarray data were normalized by the normalized quantiles function of the preprocessCore package in R software (version 3.4.1). Probes were converted to gene symbols according to the annotation information of the normalized data in the platform. Probes matching multiple genes were removed from these datasets; the average expression value of genes measured by multiple probes was calculated as the final expression value and, as in the case of the same dataset and platform but in different batches, used the removeBatchEffect function of the limma package in the R software to remove batch effects. As in different datasets or the same dataset but in different platforms, extracting multiple data sets with common gene symbols, marking different datasets or platforms as different batches, used the removeBatchEffect function of the limma package in the R software to remove batch effects. The result of the data preprocessing was assessed by Density plot. The UMAP plot was drawn to illustrate the samples before and after batch effect.

### Multi-omics data integration and analysis

To investigate the functional connections among the T2D-associated genes, we used Mergeomics, a web server for multi-omics data integration, to elucidate disease networks and predict therapeutics [9]. Mergeomics consists of two main libraries, Marker Set Enrichment Analysis (MSEA) and Weighted Key Driver Analysis (wKDA). In the current study, we used MSEA to assess whether known biological processes or pathways were enriched for multi-omics data of T2D; wKDA leverages gene network topology (interactions or regulatory relations among genes) and edge weight (strength or reliability of interactions and regulatory connections) information of graphical gene networks to predict potential key regulators of top T2D-related genes after integration. The wKDA depth was set at 1 and default incoming and outgoing directionality, the minimum overlap of 0.33, and edge factor 0.5 were used. Genes were compared against the pancreas tissue-specific Bayesian network.

Based on the key driver genes screened above, the Cytoscape (version 3.9.1) [10] software was exploited for network analysis and visualization. Here, we used 9 common algorithms (MCC, MNC, Neck, ECC, Degree, Closeness, Radiality, Stress, EPC) to evaluate modules. Subsequently, we constructed a co-expression network of these hub genes via GeneMANIA (http://www.genemania.org/) [11], which is a reliable tool for identifying internal associations in gene sets.

### Enrichment analyses of hub genes

To uncover the biological function related to the hub genes, Gene Ontology (GO) analysis and Kyoto Encyclopedia of Genes and Genomes (KEGG) pathway enrichment analysis were performed using Hiplot (https://hiplot.com.cn/).

### RNA isolation and RT‐qPCR analyses

A total of 6 serum samples (3 serum samples from T2D patients and 3 serum samples from healthy controls) were evaluated in this study. The total RNA was isolated using RNA extraction kit (TIANGEN) and reverse transcribed into cDNA using reverse transcription kit (ABI). Real-time quantitative PCR (RT-qPCR) analysis was performed using real‐time PCR kit (ABI). The relative mRNA expression levels of *PSMB9, COL1A1, COL4A1, HLA-DQB1, COL3A1, IRF7, COL5A1, CD74, HLA-DQA1,* and *HLA-DRB1*were normalized with the *GADPH* in the same sample. The thermal cycler parameters for the amplification of these genes were as follows: 1 cycle at 95 °C for 10 min followed by 40 cycles at 95 °C for 15 s, 60 °C for 15 s, and 72 °C for 30 s. Gene expression was evaluated by the 2 − ΔΔCt method. The sequences of RT‐PCR primers are the following (5′–3′; Table 2). For gene expression using RT- PCR, statistical analysis was performed by the unpaired two-tailed t-test.

**Table 2:** Basic information on RT‐qPCR analyses

	Forward	Reverse	Amplicon Size
HLA-DQA1	AGATGAGCAGTTCTACGTGGA	ACGGGAGACTTGGAAAACACT	207
HLA-DQB1	CCATCCTAAGGTGACTGTGTATCC	ATTCCACTGTGAGAGGGCTTGT	278
HLA-DRB1	ACCTTCGGGTAGCAACTGTC	AAATCCTCGGGAGAGTCTCTG	82
COL1A1	GAGGGCCAAGACGAAGACATC	CAGATCACGTCATCGCACAAC	140
COL3A1	TTGAAGGAGGATGTTCCCATCT	ACAGACACATATTTGGCATGGTT	83
COL4A1	CCAGGGGTCGGAGAGAAAG	GGTCCTGTGCCTATAACAATTCC	203
COL5A1	TACAACGAGCAGGGTATCCAG	ACTTGCCATCTGACAGGTTGA	136
PSMB9	GGAGGTCAGGTATATGGAACCC	CCTGGCTTATATGCTGCATCC	113
IRF7	CCCACGCTATACCATCTACCT	GATGTCGTCATAGAGGCTGTTG	202
CD74	GCTGGACAAACTGACAGTCAC	CAGGTGCATCACATGGTCCT	205
GAPDH	ACAGCCTCAAGATCATCAGC	GGTCATGAGTCCTTCCACGAT	104

### iTRAQ-based quantitative proteomic analysis of obese diabetic mice

All animal studies were approved by the Animal Care and Use Committee of our institution and in accordance with relevant guidelines and regulations. Five-week-old male Sprague–Dawley (SD) rats initially weighing 160–180 g were housed individually in cages at a constant temperature of 24 ± 2 °C with a 12:12-h light–dark cycle. Rats were fed a high-fat diet and intraperitoneally injected low-dose streptozotocin (32 mg/kg) to induce T2DM model. Rats with random blood glucose ≥ 16.7 mmol/L on 3 consecutive days were selected [12]. Finally, 4 rats were selected in T2D group.

Pancreas tissues were grinded and then dissolved in SDT lysis buffer (4% sodium dodecyl sulfate, 100 mM Tris–HCl, pH 7.6, Sangon, China). The supernatant was collected and quantified after boiling and centrifuging. The extracted proteins were treated with the method of filter-aided sample preparation (FASP) enzymatic hydrolysis. The samples were labeled according to the instructions of the iTRAQ Reagents 8-plex kit (AB SCIEX, USA). Mascot software 2.6 and Proteome Discoverer software 2.1 (Thermo Fisher Scientific) were used to process proteomic data against the rat database (Uniprot_RattusNorvegicus_36080_20180123).

After merging the GSE77943 (including 5 islet samples from normal mice), the comparison between the hub genes expression of T2D and contorl was performed with the T-test. P-value < 0.05 was considered significant.

### Analysis of the predictive value of biomarkers

Receiver operator characteristic (ROC) curve analysis was performed to predict the diagnostic effectiveness of biomarkers by SSPA Statistics 23. The area under the ROC curve (AUC) value was utilized to determine the diagnostic effectiveness in discriminating T2D from control samples in the GSE25724 dataset.

### Prediction and verification of transcription factors (TFs)

Transcriptional Regulatory Relationships Unraveled by Sentence-based Text mining (TRRUST) [13] is a database for predicting transcriptional regulatory networks, which contains the target genes corresponding to transcription factors (TFs) and the regulatory relationships between TFs. TRRUST currently includes two species: human and mouse, containing 8444 and 6552 TFs target regulatory relationships of 800 human TFs and 828 mouse TFs, respectively. TFs that regulate the hub genes were obtained through the TRRUST database, and an adjusted P-value < 0.05 was considered significant. Subsequently, we verified the expression levels of these TFs in obese diabetic mice with the T-test.

### Transcriptional regulation and histone modification related to hub genes based on epigenetic data

"Homo sapiens" and "pancreas islet" were jointly searched in the Cistrome Data Browser (DB) (http://cistrome.org/db) [14]. Through the analysis and processing of all samples in the preset process, as well as the evaluation using comprehensive quality control indicators, we obtained the factors targeting specific genes and visualized them in the UCSC genome browser.

### Construction of disease network

The gene-disease association networks were created using DisGeNet [15], OMIM [16], OpenTargets [17], and Genecards databases [18], and genes were selected as nodes in the network if retrieved by at least two databases using Venny2.1.0 (http://bioinfogp.cnb.csic.es/tools/venny/) [19].

### Drug repositioning

Potential drugs for the management of T2D were selected using the network based drug repositioning method from the Pharmomics pipeline in the Mergeomics web server. Drug-target interactions were used to construct a drug-target interaction network and visualized using Cytoscape v3.9.1. Gene Ontology and KEGG pathway analysis can clarify the role of potential targets by gene function and signaling pathways. The drug-disease common targets were converted into Entrez IDs, and then the “clusterProfiler” package was installed in the R software. According to the converted Entrez IDs, enrichment analysis of key target gene GO functions and analysis of KEGG signaling pathways were performed with p < 0.05.

### Molecular docking

To analyze the binding affinities and modes of interaction between the drug candidate and their targets, AutodockVina 1.2.2, a silico protein–ligand docking software was employed [1]. The molecular structures of sunitinib were retrieved from PubChem Compound (https://pubchem.ncbi.nlm.nih.gov/) [2]. The 3D coordinates of *COL1A1* (PDB ID, 5CTD; resolution, 1.6 Å), *COL4A1* (PDB ID, 1LI1; resolution, 1.9 Å)*, PSMB9* (PDB ID, 7AWE; resolution, 2.3 Å)*, IRF7* (PDB ID, 2O61; resolution, 2.8 Å)*, HLA-DQB1* (PDB ID, 1JK8; resolution, 2.4 Å) and *COL3A1* (PDB ID, 4AE2; resolution, 1.68 Å) were downloaded from the PDB (http://www.rcsb.org/pdb/home/home.do). For docking analysis, all protein and molecular files were converted into PDBQT format with all water molecules excluded and polar hydrogen atoms added. The grid box was centered to cover the domain of each protein and to accommodate free molecular movement. The grid box was set to 30 Å × 30 Å × 30 Å, and the grid point distance was 0.05 nm. Molecular docking studies were performed by Autodock Vina 1.2.2 (http://autodock.scripps.edu/).

### Statistical analysis

Statistical analysis was performed using GraphPad software (GraphPad Prism v9.0; GraphPad Software, USA) and R software (version 3.4.1).

## Results

### Multi-omics data collection and integration

We integrated the multi-omics data, including gene expression profiles and epigenomic profiling data sets collected from the GEO database (GSE38642 and GSE21232) and genomics data retrieved from *Mergeomics* web server (Table 1).

### Marker set enrichment analysis (MSEA)

To better understand the biological implications that relate to T2D, based on the multi-omic profile, we applied marker set enrichment analysis (MSEA) to evaluate the biological modules and functional categories. GO analysis results are mainly enriched in glucose homeostasis, carbohydrate homeostasis, and lipoprotein particle binding (see Additional file 1: Fig. S1A–C).

**Fig. 2:**
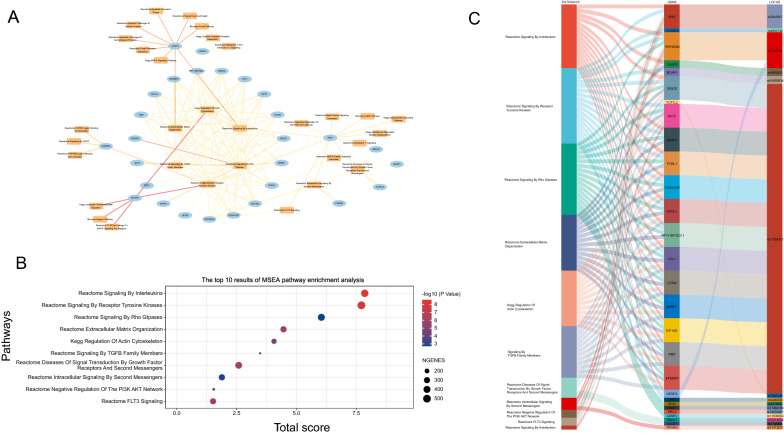
MSEA enrichment analysis of the multi-omics profile. **A** Pathway-gene network; The ellipse in the network represents hub genes, and squares represent pathways. The thickness of the line represents the MSEA score, line colors represent the number of multi-omics markers. **B** The top 10 results of MSEA pathway enrichment analysis. **C** Sankey diagram direct shows the relationship among pathways, genes, and SNPs

In order to identify the regulated modules and potential association between significant modules (FDR < 0.05 and MSEA score > 5) and genes in T2D, module-gene network were visualized (see Additional file 5: Table S1) (Fig. 2A) Of the 32 statistically significant modules, the top 10 modules are shown after applying 12 algorithms in the plug-in cyto-Hubba (Fig. 2B) (see Additional file 5: Table S2), and all 10 modules reported by MSEA are implicated in T2D. Besides, we further summarized 15 SNPs from 28 genes in the top 10 modules to investigate the aggregate genetic link between the modules and T2D (Fig. 2C).

### Weighted key driver analysis (wKDA) of T2D-related genes and analysis of hub genes

**Fig. 3:**
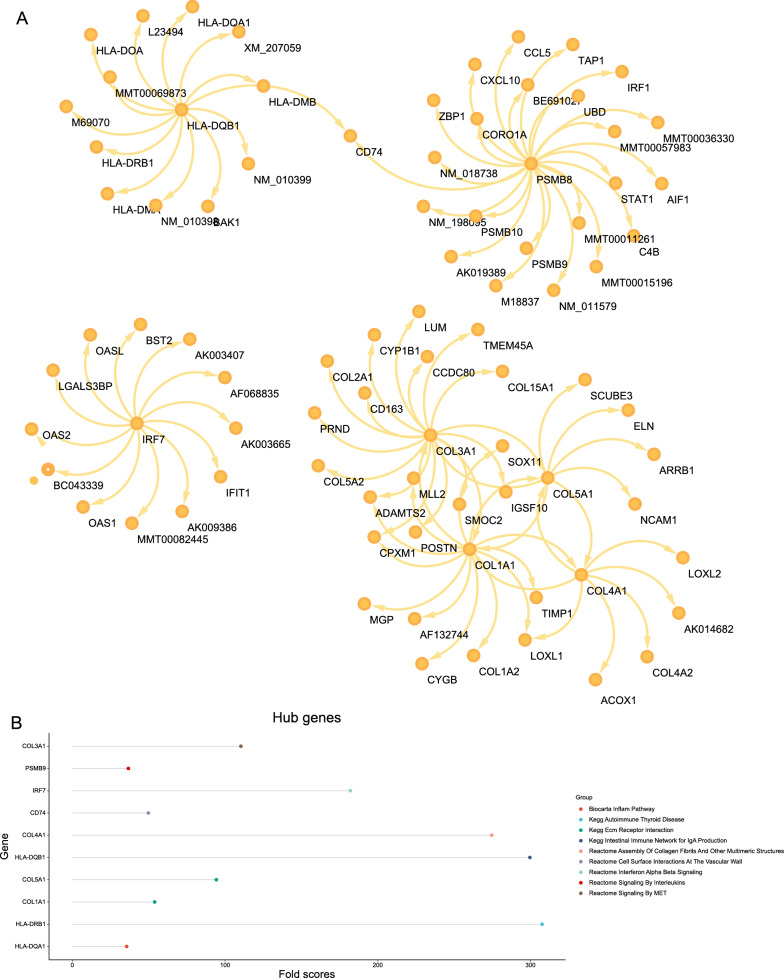

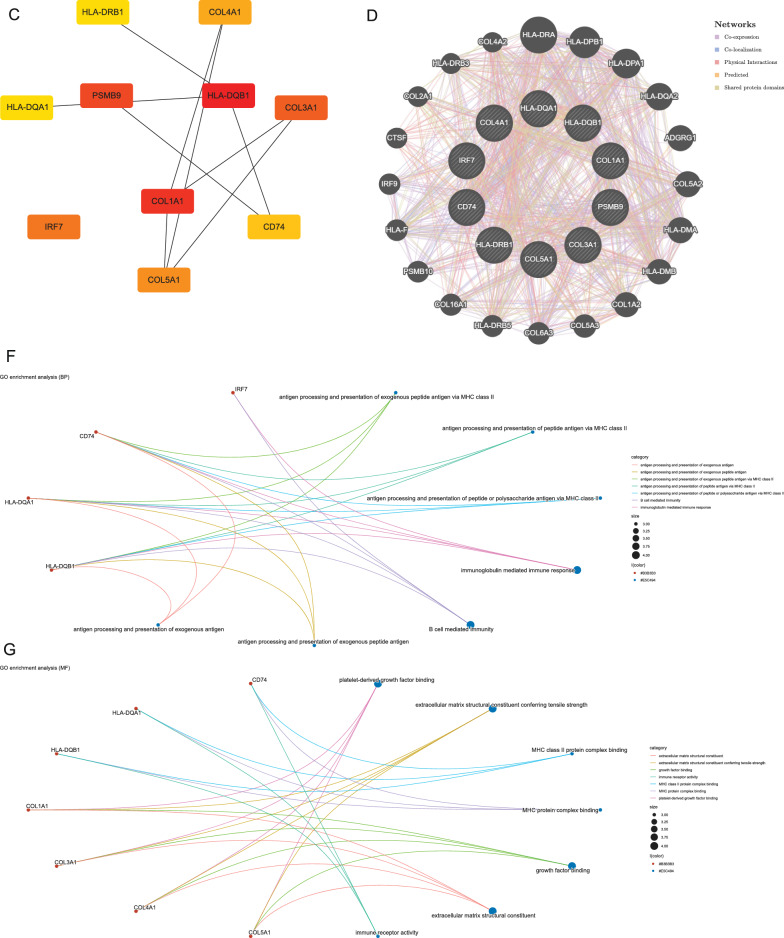

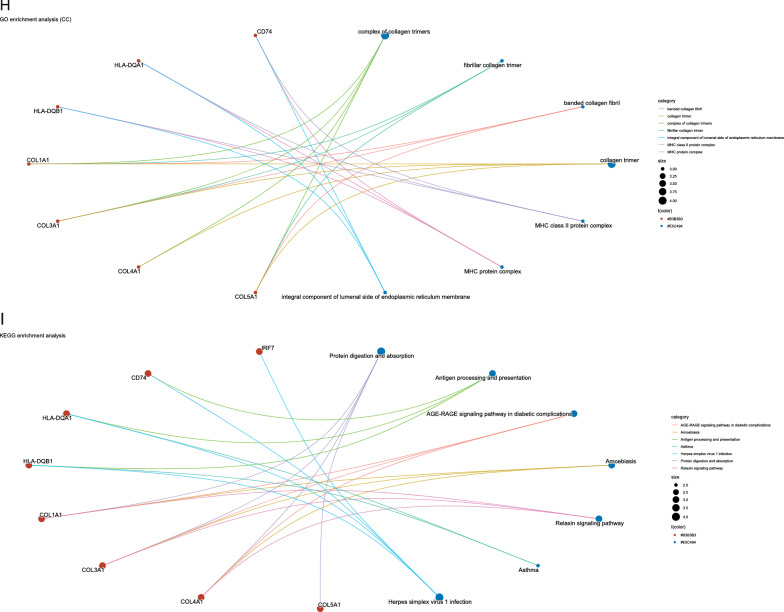
Gene subnetworks and top network key drivers (KDs) of DEGs in T2D.** A** kidney KDs and subnetworks. **B** Fold scores of top10 KDs and functional modules they belong to. **C** Key driver network of T2D. **D** Key drivers and their co-expression genes were analyzed via GeneMANIA. **E**–**I** GO and KEGG enrichment analysis of the key drivers

The wKDA using Mergeomics was used to evaluate potential networks and key regulators of type 2 diabetes-related genes based on multi-omics data. wKDA identified a network within the 84 genes (Fig. 3A). The top 10 key drives of the network were further screened (see Additional file 5: Table S3) (Fig. 3B, C), and their full names and detailed functional information are shown (see Additional file 5: Table S4). Based on the GeneMANIA database, we analyzed the co-expression network of these genes, which showed the complex PPI network with a co-expression of 35.34%, physical interactions of 22.12%, and co-localization 25.21%, shared protein domains of 8.23% and predicted of 9.10%. (Fig. 3D) GO analysis showed that these genes are mainly involved in immunoglobulin-mediated immune response, B cell-mediated immunity, immune receptor activity, and MHC class II protein complex (Fig. 3E–H). In addition, KEGG pathway analysis showed that they are mainly involved in the AGE-RAGE signaling pathway in diabetic complications and the relaxin signaling pathway (Fig. 3I).

### Validation of hub genes

**Fig. 4:**
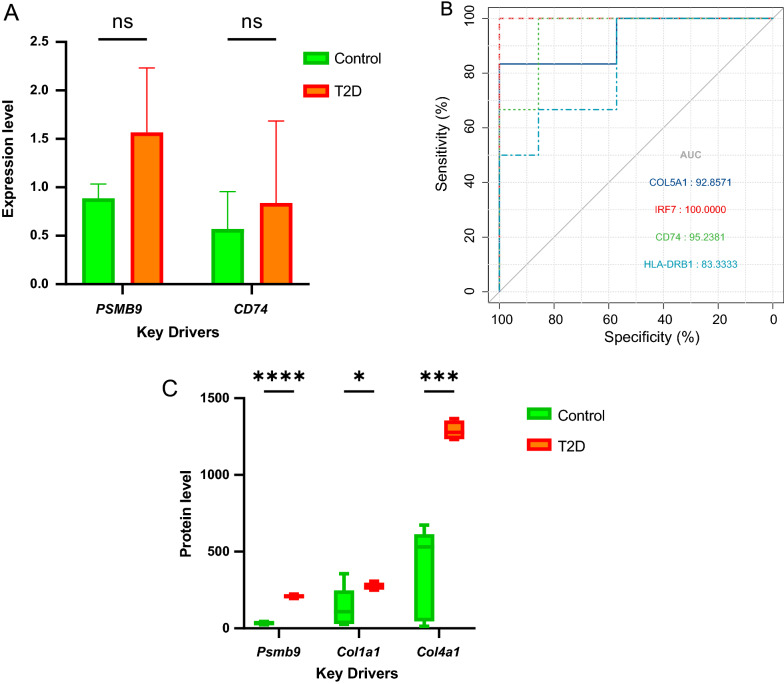
Validation of key drivers. **A** RT-qPCR analysis of the mRNA expression levels of CD74 and PSMB9 between T2D and control. **B** The GSE25724 dataset was used to validate the diagnostic effectiveness of the biomarkers for T2D by ROC analysis. **C** Protein expression of 3 key drivers (*PSMB9, COL1A1*, and *COL4A1*) in T2D and control. *P < 0.05, **P < 0.01, ***P < 0.001, ****P < 0.0001 with comparisons indicated by lines. *RT-qPCR* reverse transcription-quantitative PCR, *ns* not significant

To make the results more reliable, the expression of hub genes was subjected to RT-qPCR verification from 3 T2D patients and 3 controls. No significant difference was observed in mRNA level between the control and T2D (*PSMB9*: P = 0.16; *CD74*: P = 0.64), although there were modest trends (Fig. 4A). Besides, the GSE25724 dataset was used to validate the diagnostic effectiveness of the biomarkers for T2D by ROC analysis. (Fig. 4B) AUC of more than 0.800 was considered as having the capability to diagnose T2D with excellent specificity and sensitivity. As shown in Fig. 4B, the AUC values of *COL5A1, IRF7, CD74*, and *HLA-DRB1* were 0.928, 1.000, 0.952, and 0.833, respectively.

Further expression validation of the hub genes was performed in obese diabetic mice, which was achieved by comparing proteomics data from obese diabetic mice and data from the GEO database (GSE77943) after normalization and batch effect adjustment. The results show that expression levels of *PSMB9, COL1A1,* and *COL4A1* had significantly higher in T2D. (Fig. 4C) (The expression value of *HLA-DQB1, COL3A1, IRF7, COL5A1, CD74, HLA-DQA1,* and *HLA-DRB1* are missing in proteomics data from obese diabetic mice).

### Prediction and verification of transfer factors (TFs)

**Fig. 5:**
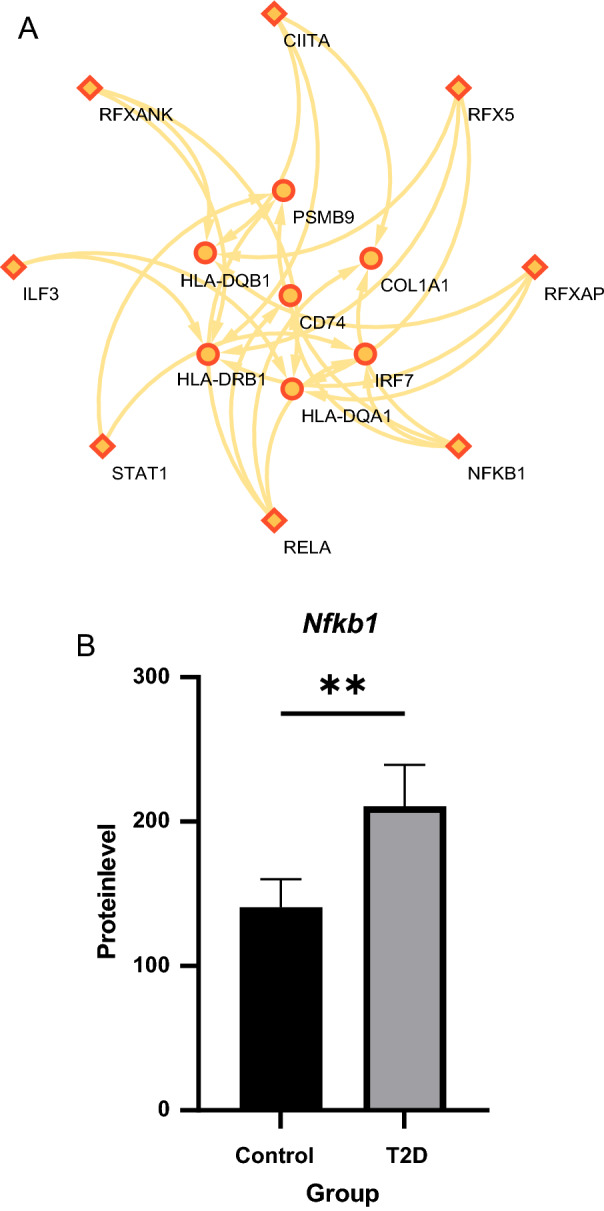
TFs regulatory network prediction and validation. **A** TFs regulatory network. TFs were marked with diamonds, and the key drivers were marked with circles. **B** Protein expression of *Nfkb1* in T2D and control. **P < 0.01

Based on the TRRUST database, we found that 8 TFs may regulate the expression of these genes (Fig. 5A) (see Additional file 5: Table S5). Further verification, we discovered that *NFKB1* is highly expressed in the T2D group (Fig. 5B) (the expression value of *RFXANK, RFXAP, RFX5, CIITA, ILF3,* and *RELA1* missing in proteomics data from obese diabetic mice), which coordinately participated in the regulation of four hub genes (*IRF7, PSMB9, CD74,* and *COL1A1*).

### Transcriptional regulation and histone modification related to hub genes based on epigenetic data

**Fig. 6:**
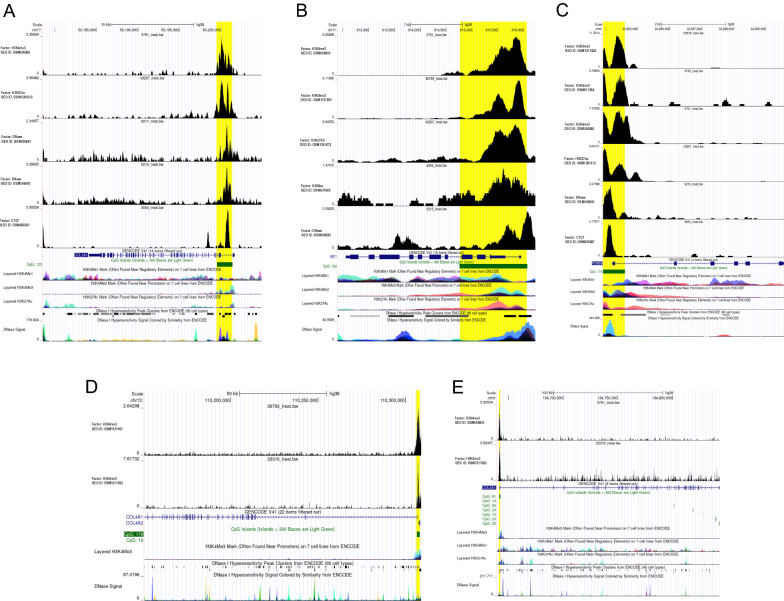
Prediction of epigenetic regulatory mechanisms of key drivers. **A**–**E** Re-analysis of different ChIP-seq and DNase-seq data available in the CistromeDB platform and the presence of DNA peaks were evaluated in *COL1A1*, *IRF7*, *PSMB9*, *COL4A1*, and *COL5A1*, respectively

After quality control analysis, ChIP-seq results showed that the *CTCF* binding site was located on the CpG islands after the first exon of *COL1A1* (Fig. 6A). In addition, *CTCF* was also combined with the promoter of *PSMB9*, which contains CpG islands (Fig. 6C). Moreover, we found that the regulation of hub genes including *COL1A1*, *IRF7*, *PSMB9*, *COL4A1*, and *COL5A1* is widely related to histone modifications such as histone methylation and acetylation (Fig. 6A–E). The modification of H3K4me3 in pancreatic islets was confirmed to depend on the presence of CpG [20], which explains our result that CTCF binds near the chromatin region occupied by H3K4me3. Concerning chromatin accessibility, highly sensitive sites of DNase I were found on *COL1A1*, *IRF7*, and *PSMB9* (Fig. 6A–C), which directly indicated the location of regions where transcriptional regulatory elements can bind.

### Drug repositioning based on multi-omics data

**Fig. 7:**
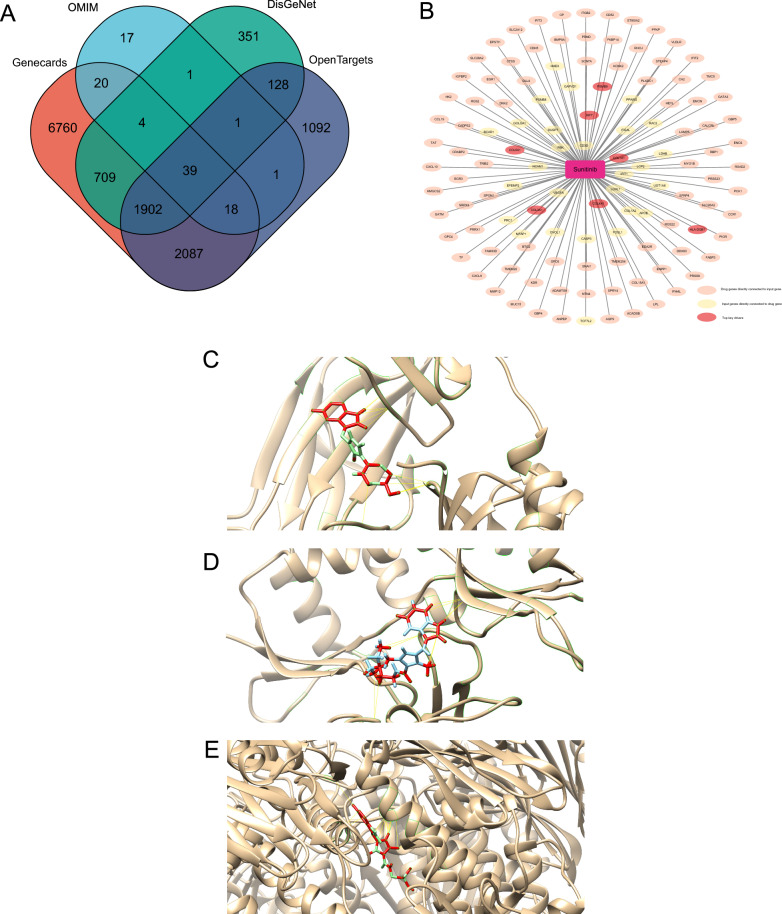

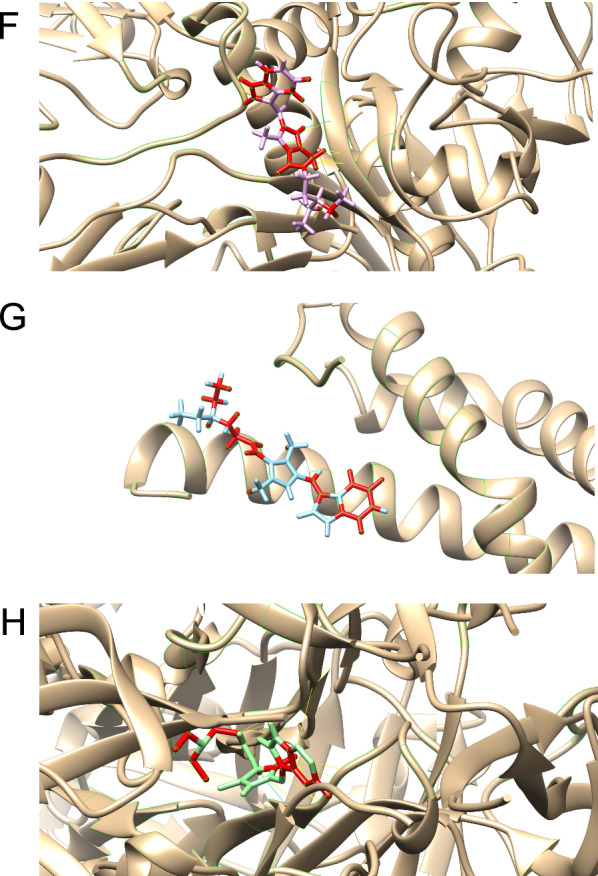
Drug repositioning and network pharmacology analysis. **A** T2D-related genes selected from *DisGeNet, OMIM, OpenTargets,* and Genecards databases. **B** Pharmacology network of the sunitinib. **C** Molecular docking simulation of sunitinib to *COL1A1*(Collagen alpha-1(I) Chain). **D** Molecular docking simulation of sunitinib to *COL3A1*(Collagen Alpha-1(III) Chain). **E** Molecular docking simulation of sunitinib to *HLA-DQB1*(MHC class II HLA-DQ8). **F** Molecular docking simulation of sunitinib to *IRF7* (Interferon regulatory factor 7). **G** Molecular docking simulation of sunitinib to *PSMB9* (Proteasome subunit beta type-9). **H** Molecular docking simulation of sunitinib to *COL4A1* (Collagen alpha-1(IV) chain)

1. Construction of multi-omics network and disease network
  We merged the MSEA network and wKDA network to construct a multi-omics network for repositioning. The gene-disease association networks were created using *DisGeNet, OMIM, OpenTargets,* and Genecards databases, and genes were selected as nodes in the network if retrieved by at least two databases (Fig. 7A).
2. T2D-targeted screening for candidate drugs
  According to the above results, a total of 1274 compounds were screened from the Mergeomics database. 17 compounds with intervention records from pancreas were proposed to possess therapeutic potential against T2D (see Additional file 5: Table S6). After excluding compounds that have not yet been widely used in clinical, sunitinib, a receptor tyrosine kinase inhibitor and chemotherapeutic agent used for the treatment of renal cell carcinoma (RCC) and imatinib-resistant gastrointestinal stromal tumor (GIST), was considered a potential drug of T2D.
3. Network pharmacology approach and molecular docking to predict the mechanisms of drugs counteracting T2D.

87 drug genes directly connected to multi-omics network and 33 genes from multi-omics network directly connected to drug genes were identified as possible anti-diabetic targets of sunitinib. (Fig. 7B) The target genes of sunitinib were further analyzed by GO and KEGG analyses (see Additional file 2: Fig S2). The results show that sunitinib may affect the AGE-RAGE pathways and gluconeogenesis, thereby influencing the development of T2D.

To evaluate the affinity of sunitinib for their targets, we performed molecular docking analysis. The binding poses and interactions of sunitinib with seven top key drivers from targets were obtained with Autodock Vina v.1.2.2 and binding energy for each interaction was generated. (The 3D-structure of the molecular target of COL5A1 was missing in the PDB database) Results showed that each target bound to sunitinib through visible hydrogen bonds and strong electrostatic interactions, moreover, hydrophobic pockets of each target were occupied successfully by sunitinib (Fig. 7C–H; Table 3).

**Table 3:** Molecular docking results of sunitinib

Gene name	Molecular targets	Estimated ΔG (kcal/mol)
HLA-DQB1	MHC class II HLA-DQ8	− 6.9
COL3A1	Collagen Alpha-1(III) Chain	− 8.7
PSMB9	Proteasome subunit beta type-9	− 7.8
COL1A1	Collagen alpha-1(I) chain	− 8.4
IRF7	Interferon regulatory factor 7	− 9.5
COL4A1	Collagen alpha-1(IV) chain	− 7.3

## Discussion

The current paper demonstrated the molecular mechanisms associated with T2D progression by combining transcriptomic, genomic, epigenetic, and proteomic data. At the same time, some less-reported genes and pathways that may play key regulatory roles in T2D have been identified, which could provide new biomarker options for T2D research. In addition, this study conducted drug repositioning based on multi-omics data to facilitate the clinical translation of potential T2D therapeutics.

To identify markers for T2D and better understand the underlying pathways, we performed MSEA using multi-omics data. In the results, GO terms were enriched in “response to hypoxia” and “response to decreased oxygen levels”, implying the stress of oxygen deprivation. Hypoxia could activate the hypoxia-inducible factor rapidly, leading to the conversion of glucose utilization from aerobic to anaerobic metabolism, resulting in β-cell dysfunction [21, 22]. Besides, Li et al. found that hypoxia may cause intensive apoptotic injury of β cells by destroying islet vascular integrity [23]. Related to the above, “gap junction assembly” and “mitochondrial outer membrane” were also enriched. β cells are connected by gap junction, which provides electric coupling between β cells, thus promoting the regulation of electrical activity and insulin secretion [24]. Studies have confirmed that related to the abnormal expression of gap junction proteins, the breakdown of mitochondrial redox balance in β cells under long-term hyperglycemia will accelerate the dysfunction of β cells [25]. On the other hand, “sulfur compound binding” was also enriched in the GO-based list. Sulfur-containing compounds include sulfur-containing amino acids, glutathione, etc. Elevated cysteine of sulfur-containing amino acids is associated with a doubling of the risk of insulin resistance [26]. In addition, data from a prospective cohort study showed that diabetic patients had higher cystathionine and plasma total cysteine and lower antioxidants such as taurine [27]. These findings suggest that sulfur-containing amino acids may interfere with blood glucose levels through oxidative stress. Moreover, the pathways “Leukocyte Transendothelial Migration” and “Reactome Signaling By Interleukins” were also significantly enriched. For islet vascular damage in diabetes, neutrophil transmigration across TNF-activated endothelial monolayers can be accelerated by co-clustering L-selectin with PECAM-1. And another crucial step is the shedding of L-selectin activated by Akt family kinases, p38 MAPK signaling pathways, etc. [28]. As previously mentioned, increased secretion and chemotaxis of neutrophils would stimulate β cell apoptosis by reducing insulin signaling transduction, promoting the occurrence of ROS-NLRP3 inflammasome-IL-1β [29].

Elucidation of T2D driving molecular profiles through integrative multi-omic analysis, including genomic, epigenomic, and transcriptomic analysis, was the primary focus of this study. The most prominent finding to emerge from the research is that putative ten hub genes are associated with T2D. Among these, *COL5A1, IRF7, CD74*, and *HLA-DRB1* expression was suggested to have diagnostic value in T2D, and the expression levels of *PSMB9, COL1A1*, and *COL4A1* were significantly higher in T2D after validation. Besides, the enrichment analysis showed the hub genes were significantly associated with immune-related terms and T2D-related terms, which corroborates the findings of a great deal of the previous work [30–32]. Chemokines and cytokines are jointly involved in the occurrence and development of T2D [33, 34], such as serum TNF-α, adiponectin, Growth factor 19/21, Interleukin-1 beta (IL-1β), Interleukin-6 (IL-6), Interleukin-18 (IL-18), and C-reactive protein (CRP), which play a central role in the development of T2D. According to KEGG analysis, the AGE-RAGE signaling pathway plays a vital role in T2D. In T2D patients, elevated blood glucose leads to advanced glycation end products [35]; the products of nonenzymatic glycation/oxidation of proteins/lipids are signal transduction ligands for Receptor for AGE (RAGE), which accumulate in the vessel wall. Furthermore, the recruitment of inflammatory cells bearing Calgranulin B (S100A9), also ligands for RAGE, augments vascular dysfunction and can subsequently exacerbate the progression of T2D [36, 37]. In addition, we found that 8 TFs may regulate the expression of these genes. We further verified that NFKB1 is highly expressed in T2D patients, which coordinately participated in regulating four hub genes (*IRF7, PSMB9, CD74,* and *COL1A1*).

In accordance with the present results, previous studies have demonstrated that *HLA-DQB1* [38]*, COL1A1* [39]*, COL3A1* [40]*, COL4A1* [41]*, CD74* [42]*, and HLA-DQA1* [38] are highly related to T2D pathogenesis. While *PSMB9, IRF7,* and *COL5A1* have not been previously reported to be associated with T2D in the literature. *PSMB9* is a multicatalytic proteinase complex with a highly ordered ring-shaped 20S core structure, which encodes a member of the proteasome B-type family, also known as the T1B family. An essential function of a modified proteasome, the immunoproteasome, is the processing of class I MHC peptides, which have critical roles in the immune response. The activation of *PSMB9* may be related to the expression of *CTCF*, which is shown in the results. *CTCF*, also called CCCTC-binding factor, encodes a transcriptional regulatory protein with 11 highly conserved zinc finger (ZF) domains. It has been revealed that CTCF-mediated chromatin accessibility changes could help to increase the transcription of genes related to important functions of pancreatic β cells, thereby increasing insulin secretion and improving T2D [43]. Diseases associated with the *PSMB9* gene in GWAS datasets from the DISEASES Experimental Gene-Disease Association Evidence Scores dataset revealed a potential association between *PSMB9* and type 1 diabetes mellitus with a standardized value of 1.20753. Besides, Phenotypes associated with the *PSMB9* gene by text-mining GWAS publications from the HuGE Navigator Gene-Phenotype Associations dataset also suggested the potential association between *PSMB9* mutations and type 1 diabetes. *IRF7* encodes interferon regulatory factor 7, a member of the interferon regulatory factor (IRF) family, which has been shown to play a role in the transcriptional activation of virus-inducible cellular genes, including interferon β chain genes. In the results of DNase-seq, the cis-acting elements located near the TSS region of *IRF7* showed high chromatin accessibility. It means that the transcription of *IRF7* may be regulated in many ways, such as H3K4me3, H3K27ac, and H3K9ac, which promote the activation of transcription. A previous study has reported that highly expressed IRF7 in the pancreas is implicated in immunoinflammatory diseases such as autoimmune pancreatitis [44] and pancreatic ductal adenocarcinoma [45]. In addition, a study by Hemin et al. demonstrated that STAT1-IRF7-MHC I complex axis was crucial for IFN-α signaling in islets and created positive feedback through IRF7-STAT2 cascade amplifying signals which accelerated the process of type 1 diabetes. *COL5A1* encodes an alpha chain for one of the low abundance fibrillar collagens. Previous work revealed that Col5A1, Nqo1, and Notch2 modulated by Ast may promote insulin-releasing balance, relieve insulin resistance, and maintain normal size in marginal-zone B cells [46]. Besides, diseases associated with *COL5A1* gene/protein from the curated CTD Gene-Disease Associations dataset suggested the potential association between *COL5A1* and diabetes mellitus with a standardized value of 1.24401 [47]. Hence, the above results provide meaningful clues for the further study of *PSMB9*, *IRF7*, and *COL5A1* in the occurrence and development of T2D, and further exploration is needed.

Due to the lack of effective/safe and less expensive drugs, drug repositioning appears to be the best tool for finding proper targets and predicting latent drugs in the therapy for T2D and related complications. Finally, 17 compounds were expected to have potential therapeutic effects on T2D. Among these, sunitinib [48] is a multi-target receptor tyrosine kinase inhibitor of vascular endothelial growth factor receptor (VEGFR), platelet derived growth factor receptor (PDGFR), etc. It is often used as a chemotherapeutic agent to treat renal cell carcinoma (RCC) and pancreatic neuroendocrine tumors. Considering that VEGF/VEGFR, PDGF/PDGFR related signal pathways play essential roles during the development of T2D, such as insulin resistance [49], the expression of iron metabolism related-proteins [50], islet cell inflammation [51], further studies to investigate potential therapeutic benefits for sunitinib in T2D are warranted.

Due to the heterogeneity of T2D [52], future T2D treatment urgently needs to be personalized. Nowadays, nanotechnology has shown great prospects in T2D pharmacological intervention, such as extending the release of anti-diabetic peptides through the hydrogel system [53], oracle delivery of nuclear acid therapy with higher stability [54], etc. In addition, the use of versatile drug delivery nanocarriers after multi-level specific biomarker recognition based on multi-omics data may increase the targeting effect of drugs and promote the realization of personalized T2D treatment [55].

Despite these promising results, questions remain. The results of RT-qPCR on the predicted hub genes could only show that the expressions of *CD74* and *PSMB9* were up-regulated in the T2D group, while the expressions of other genes were not detected (Additional files 3, 4). In addition to the poor effect of RNA extraction, since the samples used for RNA extraction were human serum rather than islets, the greater heterogeneity of the gene expression levels of the two could be the cause. For that, it would be problematic to demonstrate the expression of predicted genes in islets due to the inaccessibility of normal human islets and the potential ethical issues involved.

## Supplementary Information

**Additional file 1: Figure S1.** GO analysis of the multi-omics profile. (A-C) The results of GO were presented by bubble charts.

**Additional file 2: Figure S2.** GO and KEGG analysis of sunitinib target genes. (A-C) The results of GO were presented by bubble charts. (D) Bubble graphs showed the enrichment results of the KEGG pathways.

**Additional file 3: Figure S3.** RT-qPCR analysis of *CD74*. (A-H) The RT-qPCR process results of *CD74*.

**Additional file 4: Figure S4.** RT-qPCR analysis of *PSMB9*. (A-H) The RT-qPCR process results of *PSMB9*.

**Additional file 5: Table S1**: MSEA top pathways. **Table S2**: Scores of pathways after applying 12 topological algorithms. **Table S3**: Detailed information of key drivers in wKDA network. **Table S4**: The details of hub genes. **Table S5**: The details of TFs. **Table S6**: Detailed results of drug repositioning results.

## Data Availability

The original contributions presented in the study are publicly available. The data can be found here: https://www.ncbi.nlm.nih.gov/geo/query/acc.cgi?acc=GSE38642, https://www.ncbi.nlm.nih.gov/geo/query/acc.cgi?acc=GSE25724, and https://www.ncbi.nlm.nih.gov/geo/query/acc.cgi?acc=GSE21232.

## Abbreviations

T2D: Type 2 diabetes
GEO: Gene expression omnibus
DEGs: Differentially expressed genes
MSEA: Marker set enrichment analysis
wKDA: Weighted key driver analysis
GO: Gene ontology
KEGG: Kyoto Encyclopedia of Genes and Genomes
RT-qPCR: Real-time quantitative PCR
TRRUST: Transcriptional regulatory relationships unraveled by sentence-based text mining
TFs: Transcription factors
ROC: Receiver operator characteristic
AUC: Area under the ROC curve
IL: Interleukin
RAGE: Receptor for AGE
IRF: Interferon regulatory factor
