# Correction: Uncovering the gene regulatory network of type 2 diabetes through multi-omic data integration

**DOI:** 10.1186/s12967-023-04021-w

**Published:** 2023-03-20

**Authors:** Jiachen Liu, Shenghua Liu, Zhaomei Yu, Xiaorui Qiu, Rundong Jiang, Weizheng Li

**Affiliations:** 1 Department of General Surgery, Third Xiangya Hospital Central South University, No. 138 Tongzipo Road Yuelu District, Changsha, 410013 Hunan People’s Republic of China; 2 Department of Thyroid and Breast Surgery, The Frist Afflicted Hospital of Fujian Medical University, No. 20 Chayzhong Road, Taijiang District, Fuzhou, 350005 Fujian People’s Republic of China; 3 Xiangya Medical College, Central South University, No. 138 Tongzipo Road Yuelu District, Changsha, 410013 Hunan People’s Republic of China; 4 The Center of Systems Biology and Data Science, School of Basic Medical Science, Central South University, Changsha, Hunan People’s Republic of China

## Correction: Journal of Translational Medicine (2022) 20:604 https://doi.org/10.1186/s12967-022-03826-5

Following publication of the original article [1], we have been notified that the affiliation of one of the authors was assigned incorrectly. Correct affiliation should be as per below:

Weizheng Li 1—Department of General Surgery, Third Xiangya Hospital Central South University, No. 138 Tongzipo Road Yuelu District, Changsha 410013, Hunan, People’s Republic of China.
