# Supplementary figures and images for: Uncovering the gene regulatory network of type 2 diabetes through multi-omic data integration
Source: J Transl Med. 2022 Dec 16;20:604. doi: 10.1186/s12967-022-03826-5 (PMC9756634; doi:10.1186/s12967-022-03826-5)

A

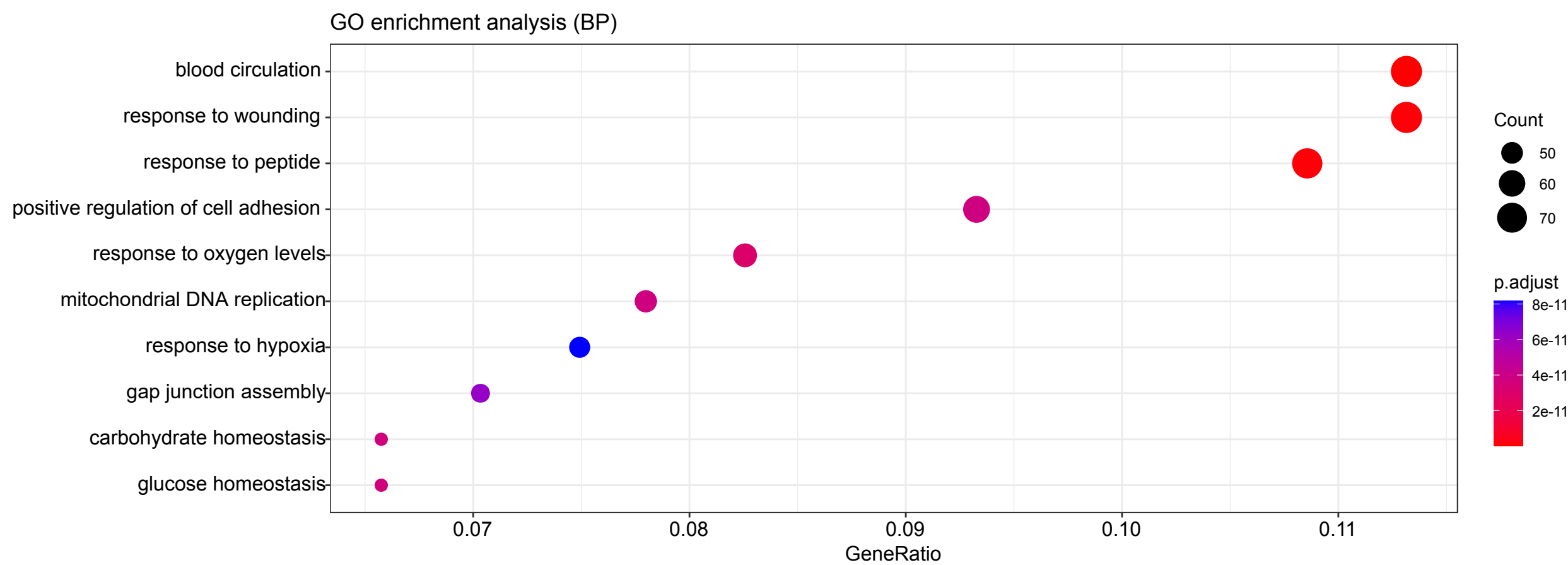

B

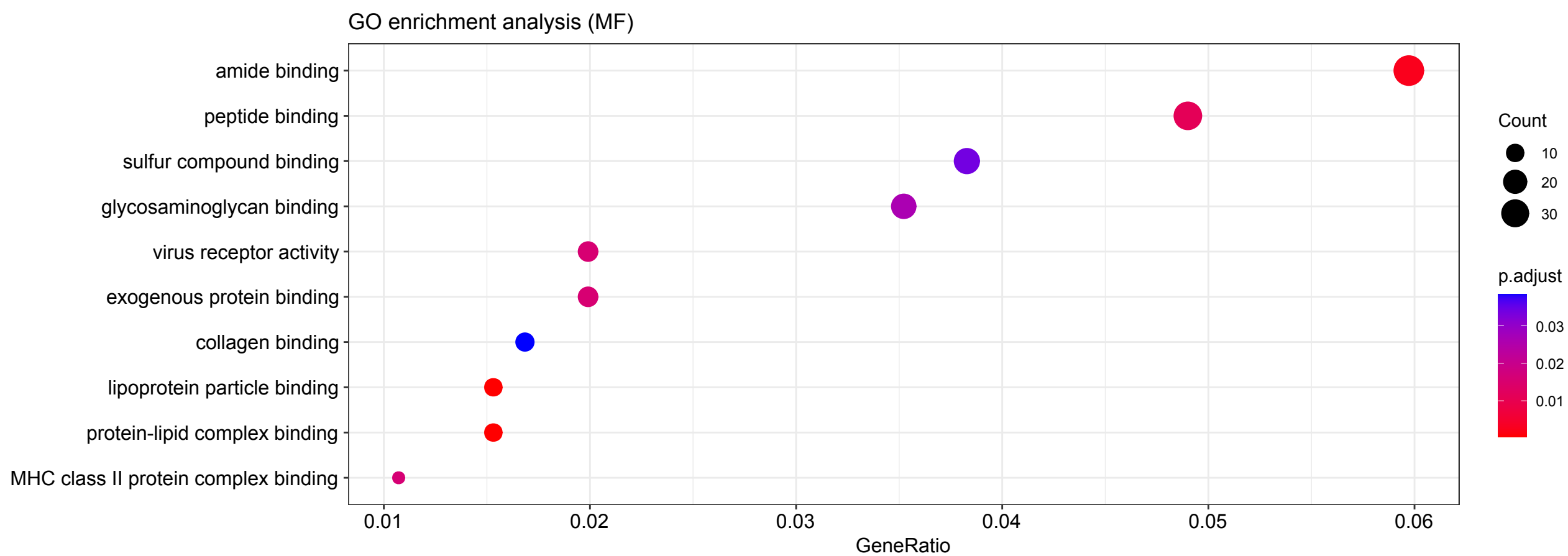

C

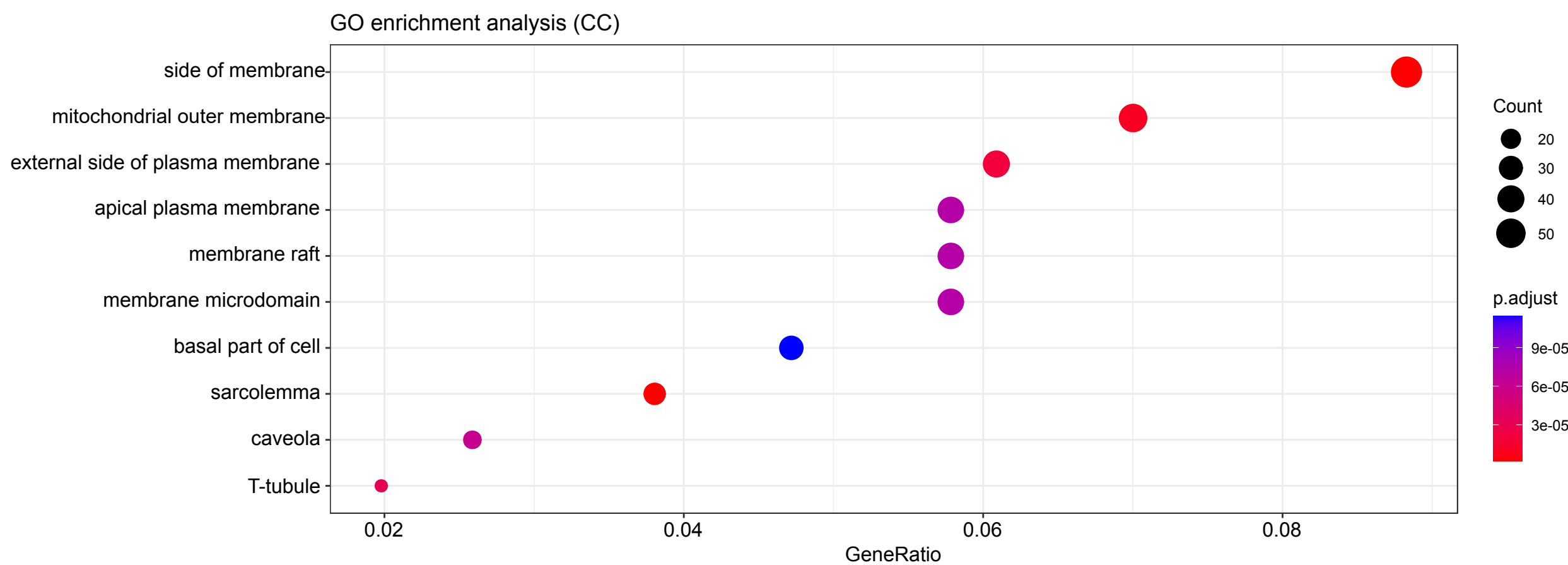

Supplement: Supplementary file 1 — Additional file 1: Figure S1. GO analysis of the multi-omics profile. (A-C) The results of GO were presented by bubble charts. [file 12967_2022_3826_MOESM1_ESM.pdf]

A

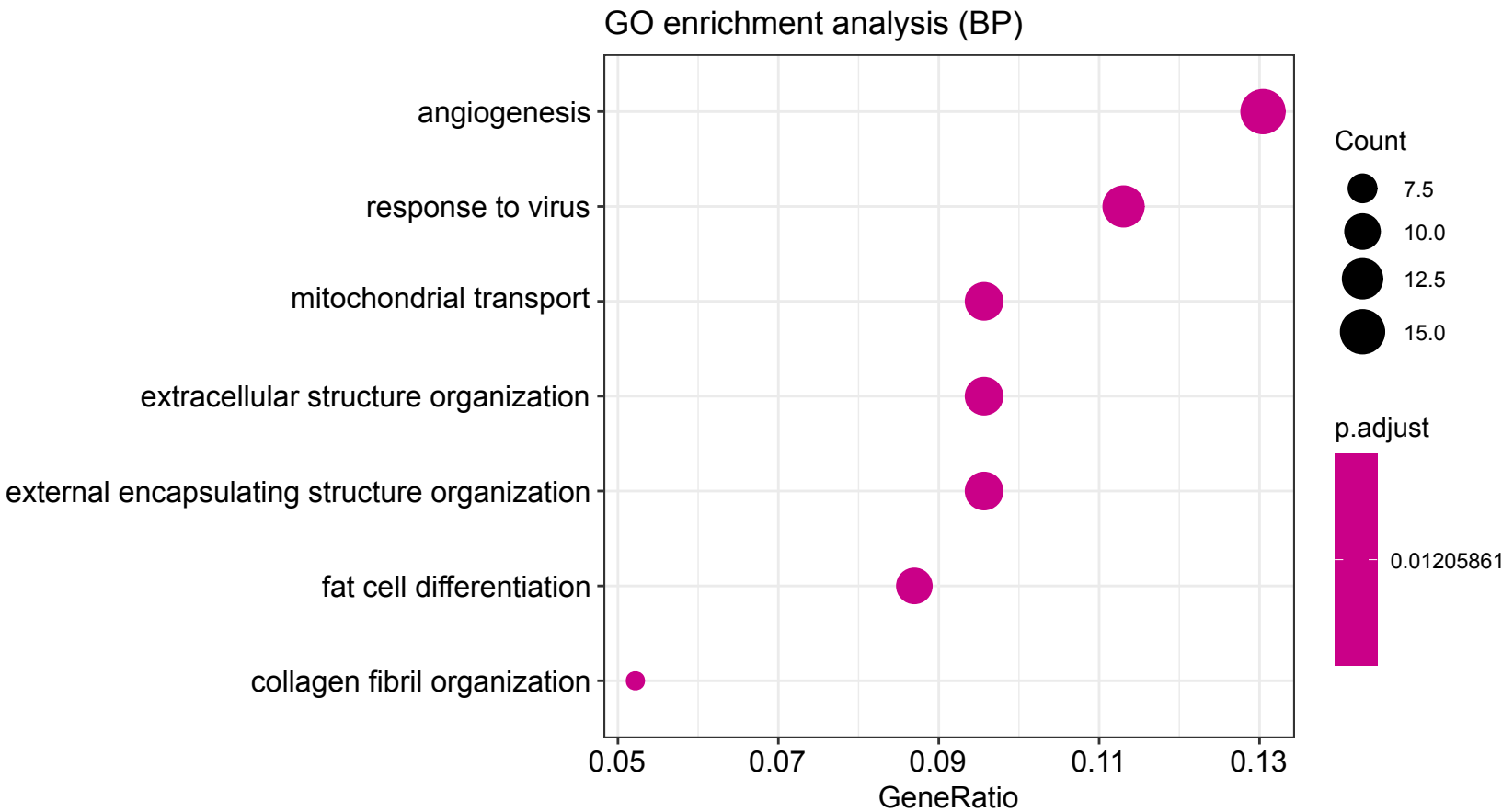

B

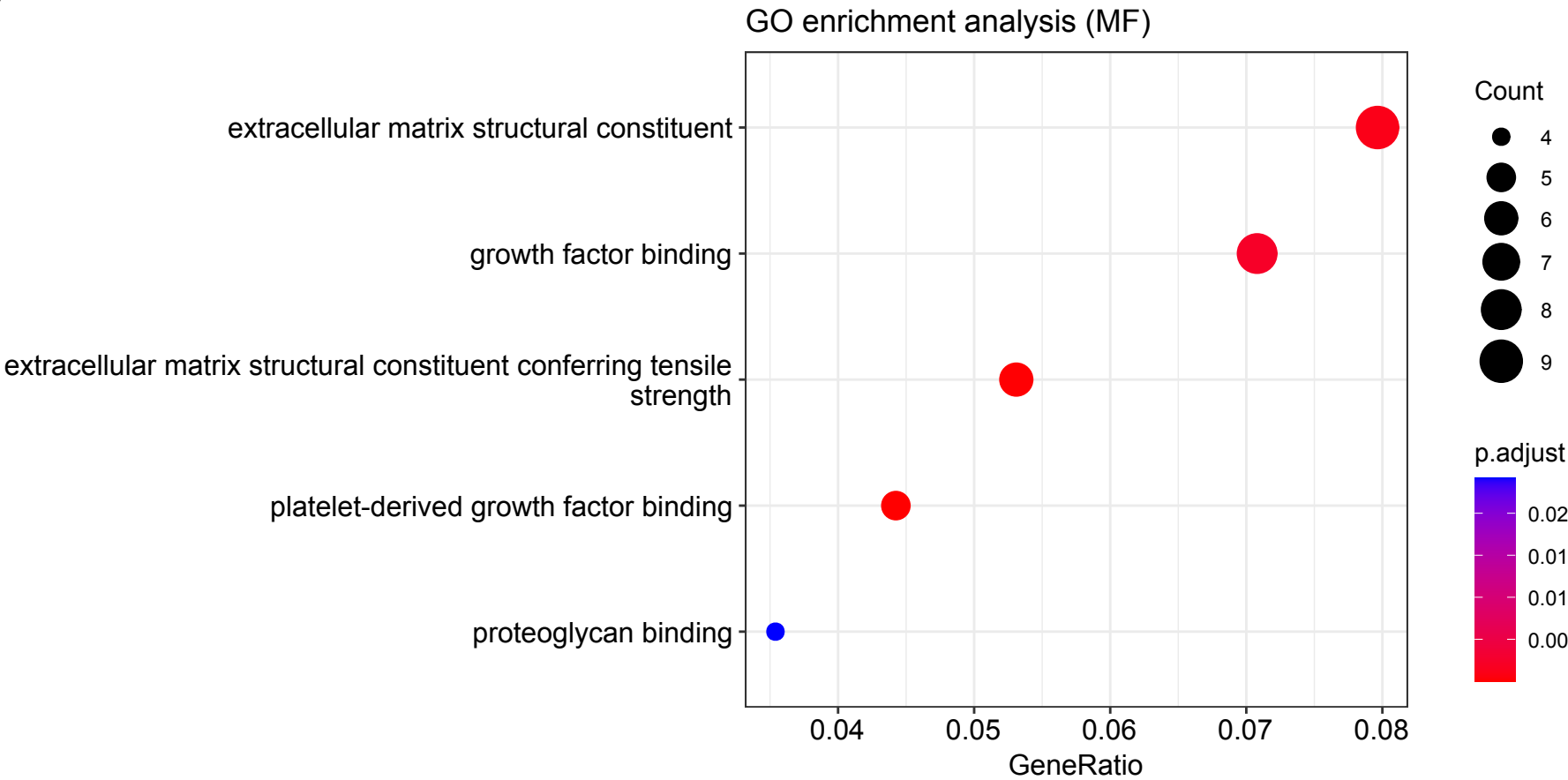

C

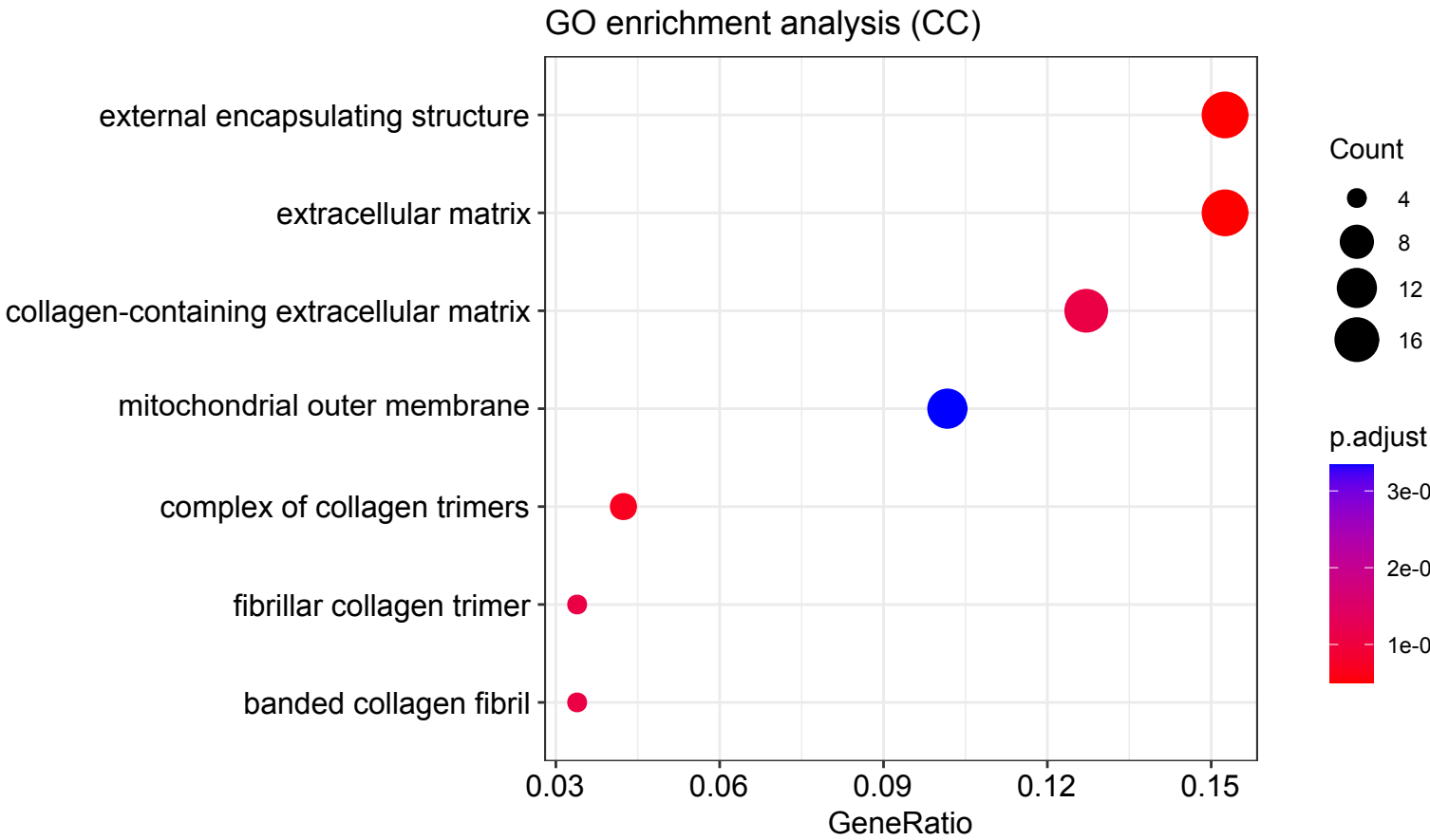

D

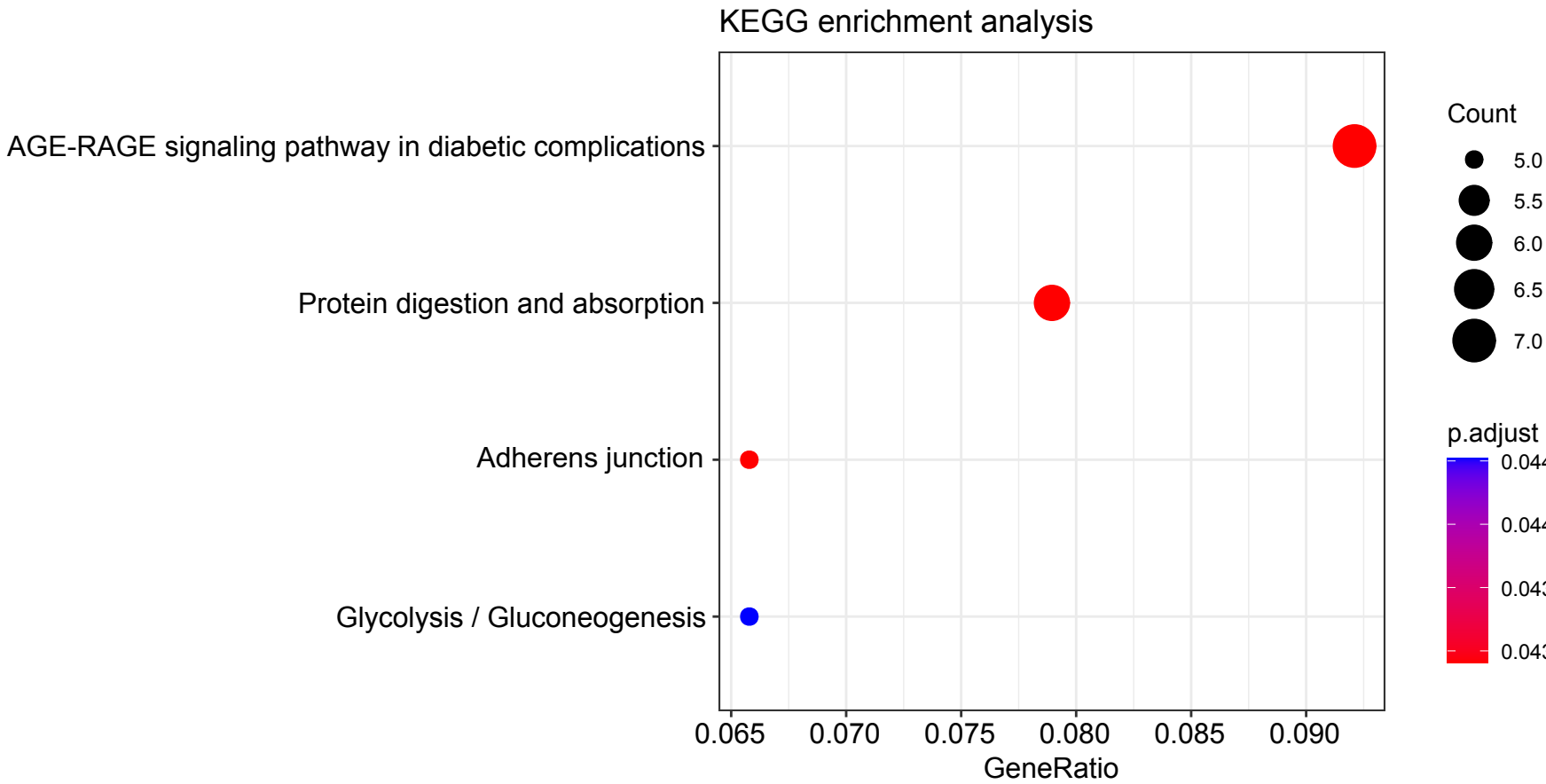

Supplement: Supplementary file 2 — Additional file 2: Figure S2. GO and KEGG analysis of sunitinib target genes. (A-C) The results of GO were presented by bubble charts. (D) Bubble graphs showed the enrichment results of the KEGG pathways. [file 12967_2022_3826_MOESM2_ESM.pdf]

A

GOI - SybrGreen

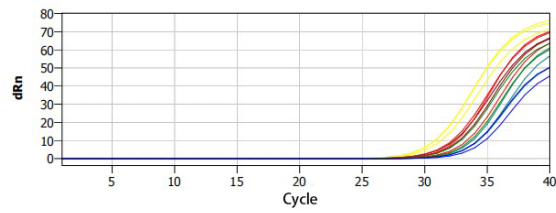

B

GOI - SybrGreen

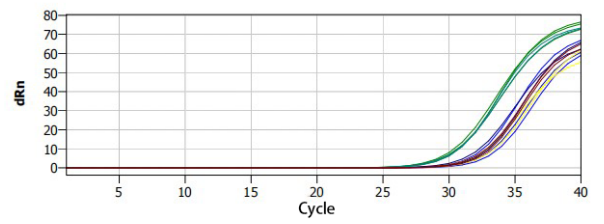

C

DIF

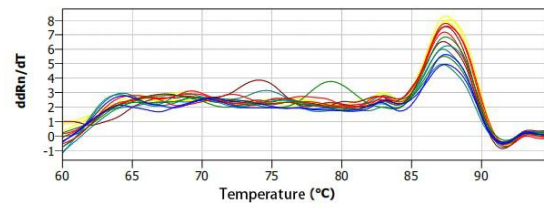

D

DIF

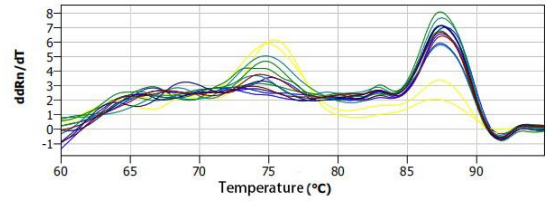

E

Ref. gene - SybrGreen

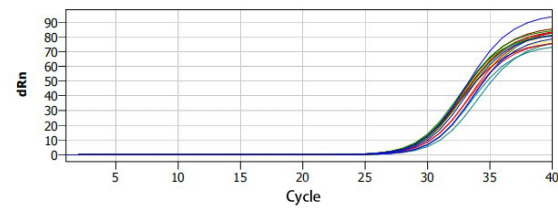

F

Ref. gene - SybrGreen

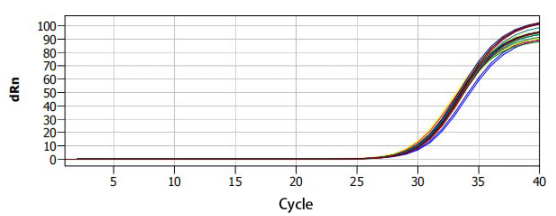

G

DIF

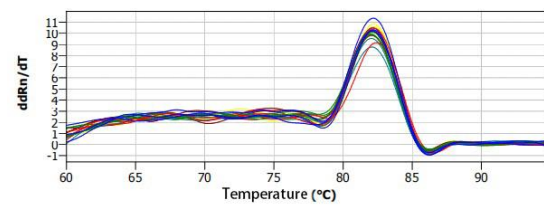

H

DIF

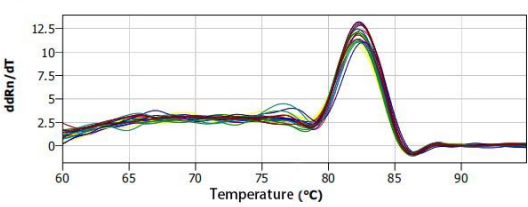

Supplement: Supplementary file 3 — Additional file 3: Figure S3. RT-qPCR analysis of CD74. (A-H) The RT-qPCR process results of CD74. [file 12967_2022_3826_MOESM3_ESM.pdf]

A

GOI - SybrGreen

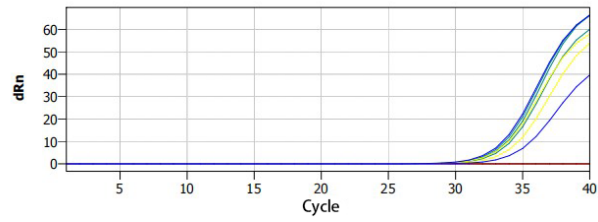

B

GOI - SybrGreen

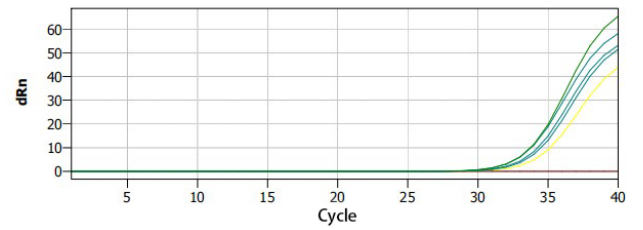

C

DIF

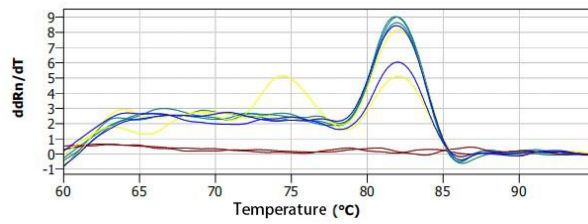

D

DIF

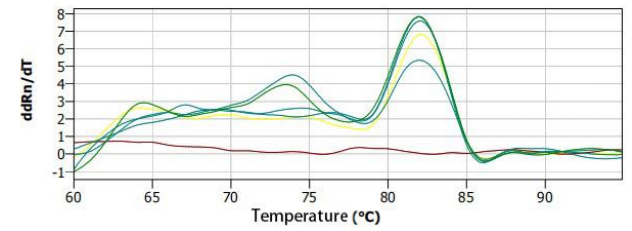

E

Ref. gene - SybrGreen

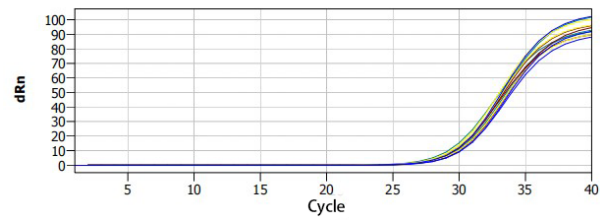

F

Ref. gene - SybrGreen

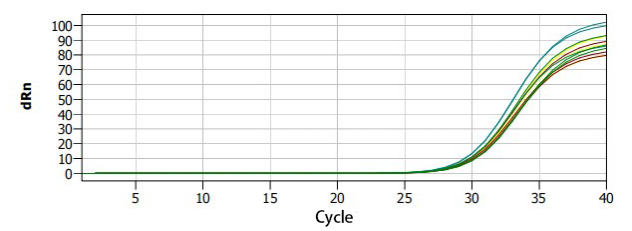

G

DIF

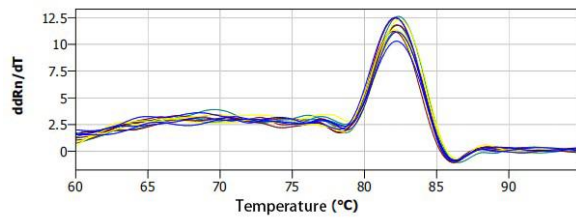

H

DIF

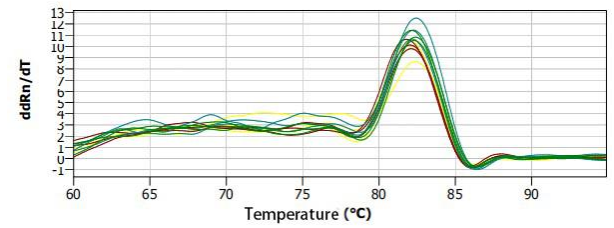

Supplement: Supplementary file 4 — Additional file 4: Figure S4. RT-qPCR analysis of PSMB9. (A-H) The RT-qPCR process results of PSMB9. [file 12967_2022_3826_MOESM4_ESM.pdf]
